# Experiences and impact of moral injury in human trafficking survivors: a qualitative study

**DOI:** 10.1186/s40359-024-02157-y

**Published:** 2024-11-14

**Authors:** Victoria Williamson, Dominic Murphy, Cornelius Katona, Christina Curry, Ella Weldon, Neil Greenberg

**Affiliations:** 1 Institute of Psychology, Psychiatry and Neuroscience, King’s College London, 10 Cutcombe Road, London, SE5 9RJ UK; 2 University of Bath, Bath, BA2 7AY UK; 3 Research Department, Tyrwhitt House, Combat Stress, Leatherhead, Surrey, UK; 4 Helen Bamber Foundation, London, NW1 0TF UK

**Keywords:** Moral injury, Human trafficking, PTSD, Trauma

## Abstract

**Background:**

Research in recent years has increasingly highlighted the relationship between exposure to potentially morally injurious events (PMIEs) and poor mental health outcomes. Human trafficking survivors often report exposure to many traumatic and PMIEs and given the complexities of trafficking exploitation, survivors may be especially vulnerable to moral injury. Despite this, no research has investigated experiences of PMIEs and moral injury in human trafficking survivors. The objective was to explore survivors’ experiences of PMIEs, the impact of PMIEs on wellbeing and functioning and the factors that may influence outcomes following PMIEs.

**Method:**

Participants were seven human trafficking survivors from diverse backgrounds who had settled in the UK. In-depth semi-structured interviews were conducted via telephone. Data were analysed using thematic analysis.

**Results:**

Human trafficking survivors were found to experience multiple PMIEs, including transgressive acts committed by others and betrayal by trusted loved ones or those in positions of authority. Experiencing PMIEs contributed towards considerable psychological distress, including intense feelings of shame, anger and worthlessness, and negatively impacted survivors daily functioning. Formal support, especially practical help (e.g. warm clothing) and social support groups, were experienced as particularly beneficial.

**Conclusions:**

This study provides some of the first evidence that human trafficking survivors may be vulnerable to moral injury and indicates the impact that exposure to PMIEs can have on functioning. Future work is needed to ensure that statutory organisations consider the potential for causing moral injury when interacting with human trafficking survivors and clinical care teams are equipped to provide tailored guidance and support.

## Introduction

Human trafficking refers to circumstances of exploitation where an individual cannot refuse or leave due to intimidation, coercion, or abuse of power [1]. In 2016 an estimated 40.3 million individuals were victims of human trafficking into different forms of exploitation globally [2]. In the last five years, this figure has grown exponentially & estimates now stand at 50 million individuals [1]. The global challenge of human trafficking has also been made more pressing due to increased economic hardship, the COVID-19 crisis, and international armed conflict, which have exacerbated the underlying drivers of all forms of human trafficking and modern slavery [1].

Trafficked individuals often encounter a range of substantial risks to health, including physical & sexual violence and high-risk living conditions [3]. Studies have documented that survivors often experience a range of adverse physical and mental health problems [4], including high levels of depression, anxiety and post-traumatic stress disorder (PTSD). Studies have also found that trafficking survivors have notably high rates of mental ill health comorbidity [5, 6]. Access to appropriate care, both psychological and physiological, has been found to be extremely limited [7]. Many trafficking survivors report facing considerable barriers to help-seeking and care including homelessness, destitution, legal stressors, guilt and shame as well as high levels of mistrust of officials, including health providers [8]. For those survivors who do successfully access psychological care, there is limited evidence available on which therapies may best treat their post-traumatic sequelae [6]. Providing psychological treatment to trafficking survivors may also be especially challenging for clinical care teams as survivors have often experienced multiple traumatic events and may suffer with complex trauma sequelae as a result.

Another challenge for clinicians can be the nature and complexities of the trafficking exploitation experienced [4, 5, 7] and it is possible that human trafficking survivors may report potentially morally injurious events (PMIEs) [9, 10]. PMIEs are typically thought to include acts of betrayal [11](e.g. a ‘boyfriend’ who grooms and manipulates a young woman into exploitative sex work), omission (e.g. a trafficked individual may witness their traffickers harshly punishing others and be unable to intervene), and commission (e.g. a trafficked individual may be forced to recruit others). The act of being trafficked itself can be morally injurious, for example a trafficked person can be knowingly or unknowingly provided with false documents whilst in the control of someone else and the knowledge that they have committed a crime can prevent them from seeking help. Exposure to PMIEs can lead to the development of moral injury, which refers to the intense distress one can experience after an event(s) that challenge one’s moral or ethical code [9]. Moral injury is characterised by profound feelings of guilt, shame, anger, and disgust and has been conceptualised as a substantial breaking down of an individual’s relationship with self and others [12]. Individuals who suffer with moral injury often report negative cognitive (e.g. ‘I am worthless,’ ‘other people cannot be trusted’) and behavioural changes (e.g. social withdrawal, problematic coping strategies). While moral injury is not a mental illness, these changes can contribute towards the development of mental health problems, including PTSD, depression and suicidality [13, 14]. Experiencing moral injury has also been linked to complex PTSD (CPTSD), and those who report more severe moral injury have been found to endorse greater CPTSD symptoms [15]. Should human trafficking survivors experience moral injury, this may present significant challenges for clinical care teams as moral injury-related mental health difficulties have been found to be notably difficult to treat [16, 17]. Standard PTSD treatments (e.g. trauma focused cognitive behavioural therapy [TF-CBT]) do not appear to fully address morally injured patients’ symptoms of guilt and shame [18] and it has been argued that some exposure-based treatments (e.g. Prolonged Exposure) may worsen symptoms [19].

The suffering human trafficking survivors experience does not often end shortly after their escape or release from exploitation. After release or escape, survivors are often then received and/or detained by governmental authorities [20]. In the UK, individuals who are identified by officials as having potentially experienced human trafficking are referred into the National Referral Mechanism scheme where for at least 30 days they can access support such as legal advice and accommodation while their case is considered [21]. However, despite the existence of this scheme, many trafficked individuals can face repeated, intensive questioning by often disbelieving police or Home Office officials; experience lengthy detention at (poorly maintained) immigration centres; and, as they are not permitted by the Government to be gainfully employed, they must survive on the Government’s allowance (which can be as little as £8.86 per person per week [22]) until a decision is reached on their case. Treatment by statutory organisations that are purportedly a source of protection and care could add to the distress human trafficking survivors experience [23] and could arguably potentially be experienced as morally injurious.

At present, it is unclear whether, or to what degree, human trafficking survivors experience moral injury. A deeper understanding of human trafficking survivors’ experiences of, and responses to, moral injury may inform clinical practice and ensure that appropriate support and guidance are available in future. To address this evidence gap, we conducted in-depth qualitative interviews with human trafficking survivors residing in the UK to explore responses to PMIEs and the impact of such events on wellbeing.

## Method

This study received ethical approval from King’s College London Research Ethics Committee (HR-20/21-18272) in accordance with the Declaration of Helsinki. All participants gave verbal audio-recorded consent for participation.

### Recruitment

Between October 2021-November 2023, eligible survivors of human trafficking were recruited to the study from the Helen Bamber Foundation (HBF). The HBF is a not-for-profit organisation that provides psychological and wellbeing support to survivors of human trafficking in London, UK (https://www.helenbamber.org/).

The present study is experiential in focus, and as experiences of PMIEs and moral injury in this population are under-researched, we prioritised sample specificity when considering the ‘informational power’ [24] of our sampling procedure and approach. That is, we aimed to incorporate in-depth insights, rather than a broader range of survivor perspectives. To be eligible to participate, participants had to be aged 18 years or more; be identified as a victim of trafficking by statutory or voluntary agencies; were no longer in an exploitative setting; have either previously received or be currently receiving post-trafficking support from non-governmental organisations; and be considered by the clinical care team at HBF to have engaged with stabilisation treatment and judged as able to tolerate the interview process.

Eligible participants were approached for participation by their clinical care team at the HBF. The HBF support workers judged the potential risks of participation and, if deemed safe, described the study aims and procedures; the nature of informed consent, and any queries/concerns participants may have regarding their participation in the study. The voluntary nature of participation was emphasised during all contacts. The contact details of eligible participants who were interested in taking part were provided to the lead researcher (VW) who contacted them to discuss the study and schedule the interview. As this study began during the social restrictions of the COVID-19 Pandemic, all study interviews were conducted via telephone, with an interpreter if required (*n* = 1 participant required an interpreter).

HBF clinicians and caseworkers identified *n* = 33 individuals as being potentially eligible for the study. Out of those approached, *n* = 12 refused to participate, *n* = 10 were unable to be contacted, and *n* = 4 were initially interested but later became unavailable or unreachable. The participants who provided a reason for refusal felt that their experiences were too distressing to talk about, did not want to be reminded of the past, or were uncomfortable with sharing their experiences with an unfamiliar person. Seven participants were successfully recruited to the study. Participants received a £20.00 shopping voucher as a thank you for their time.

### Data collection

Interviews were carried out by VW (experienced qualitative researcher) and lasted for 50 min on average (8.31 SD) (range 36–60 min). Any potentially identifying information was removed from interview transcripts and participant contact details were destroyed by the research team following participation. The participants were not known to the core researchers (VW, DM, NG) prior to interview. The interview schedule was informed by the research questions, previous qualitative moral injury research with other populations, consultation with clinicians (CK, CC) and service providers who provide treatment to patients with moral injury and human trafficking survivors, and the broader literature on responses to moral injury and trauma. The interview schedule focused on participants experiences of moral injury, the impact of PMIEs on their daily functioning and wellbeing, and whether any factors may make some human trafficking survivors more (or less) vulnerable to psychological difficulties after PMIE exposure.

Participants were not given a formal, academic definition of moral injury or PMIEs (e.g [12]). prior to the interview. Consistent with previous studies of PMIEs and moral injury, participants were asked whether they had experienced an event(s) that ‘challenged their sense of right and wrong, or their view of who they are and the world they live in’ [25, 26]. Interview questions were open-ended, allowing participants to describe their experiences in their own words [27, 28]. Prior to the interview, participants were asked to provide basic demographic information. Study interviews adhered to WHO guidance on ethical and safety recommendations for interviewing trafficked people [29] and were conducted by a trained researcher (VW), facilitated by independent interpreters if required. Throughout the study, the research team repeatedly emphasised that participation in the study was voluntary, participants could stop taking part at any time and could withdraw their data. Participants were advised that the study was not connected to any immigration or policing processes [30], and explained participation (or non-participation) would not impact on any support available from the HBF or other organisations. Participants were all provided with the opportunity to give feedback about their experience of the interview following participation. All participants were debriefed following the interview, with information about available sources of support. No adverse or safeguarding events were experienced.

### Data analysis

All transcripts were analysed using Nvivo 14 (QSR International). Interviews were analysed by the lead researcher (VW) using Thematic Analysis, following the steps suggested by Braun & Clark [31] These steps included familiarisation with the data, generating initial codes, and creating early themes, and refining core themes. An inductive analytical approach was used [28, 31, 32]. Data collection and analysis took place simultaneously, allowing topics of interest to be explored in subsequent interviews and to determine whether thematic saturation had been reached. Peer debriefing was used, with feedback on codes and themes sought from co-authors who have expertise in supporting survivors of human trafficking, moral injury and qualitative methods (NG, DM, CK, CC, EW). To ensure reflexivity, a reflexive journal was kept by the lead researcher (VW) to reflect on the influence of their own experiences, beliefs and assumptions to prevent premature or unduly biased interpretations of the data [33].

## Results

### Demographic information

Of the seven participants, five were female and the mean age of the sample was 38.86 years (SD 8.75). Five had been trafficked from African countries, while one had been trafficked from Europe, and another from Southeast Asia. Most reported being single (*n* = 6) and five reported having children. All reported having sought asylum or leave to remain in the UK.

### Qualitative findings

Three core themes were developed reflecting: (i) survivors experiences of PMIEs both during and after their ordeal of human trafficking; (ii) the impact of such events on wellbeing and functioning; and (iii) support for moral injury-related distress following PMIEs. Anonymised excerpts have been provided below.

### Experiences of morally injurious events

Morally injurious experiences primarily related to transgressive events committed by others and experiences of betrayal. The PMIEs reported often reflected multiple, ongoing processes (rather than single incidents) and included survivors’ experiences of extreme suffering at the hands of those who trafficked them, such as experiencing torture; being repeatedly threatened; having food withheld; and being forced to break religious covenants. Betrayal events included being lied to by trusted agents or family friends about prospective employment or education opportunities overseas, while other participants described being betrayed and trafficked by boyfriends. Some participants also described how the people who helped them leave their exploitative circumstances went on to subsequently exploit and traffic them in turn.

*“I’m never good with friends. I don’t trust anybody. Because it’s friends that actually mislead me to why I was a victim. It’s friends who actually mislead me to that point whereby I now become a victim of trafficking. So*,* I don’t trust anybody anymore… When we were in [my home country*,* my friend] she was the one that said she want to introduce me to a nice man. We went out and… at the end of the day*,* I was deceived*,* and I’ve been a victim.” (Female*,* 001)*.

Survivors described that they were often unable to leave or escape the trafficked situation as their traffickers drugged them, used existential threats (e.g. via the misuse of cultural practices such as voodoo), or threatened that their families would be hurt should they attempt escape. In some cases, participants described that their family members had been badly injured or killed by their traffickers to ensure the survivor’s continued cooperation or as punishment for attempting to escape. Other survivors described that they were prevented from leaving as they were held in a remote, unknown location, or their families were not financially able to secure their freedom.

*“Because I was so very young… so my mother’s friend and sister came… to talk to my mother that she want to help me to travel to abroad… they told my dad…that I’m going to further my education…. As soon as I get to [Europe] she was just bringing men to me… When I refused*,* she beat me up*,* then she used Voodoo on me*,* and she said I’m going to pay her some amount of money. So*,* she and her boyfriend was threatening me that if I tell anybody I’m going to die and they are going to kill me*,* kill my mother… when I tried to escape after one year*,* they went and beat my mum up*,* my mum later died in the hospital.” (Female*,* 002)*.

Survivors considered that, from their perspective, experiences of human trafficking were common and widespread, with poor people and women being especially vulnerable to trafficking. At the same time, traffickers were considered to be immune to any consequences, they were likely to continue to entrap others and face no repercussions for their actions. This could potentially reflect a PMIE not only in the perpetration behaviour by traffickers, but also a possible betrayal PMIE as statutory services (e.g. police) were perceived as unlikely to hold traffickers to account.

*“Where we came from people can just do whatever they want*,* they target people with children*,* they are going to do it and they are going get away with it. We’ve been threatened not to say anything… this normally happens more to women. Women are the ones that are not really safe.” (Female*,* 001)*.

#### Experiences of betrayal by authorities

Participants also reported experiencing challenging and potentially morally injurious interactions with authority figures, such as the police, immigration and Home Office staff. Some participants described trying to tell staff at UK airports of their circumstances and ask for help, which then reportedly resulted in their being detained in immigration centres for several months. Others recounted how they had not been provided with appropriate interpreters during their interviews with Home Office staff and were repeatedly accused of non-cooperation when they could not effectively communicate. Participants also described that, despite their entitlement to support, the government accommodation they were provided (or detained) to live in, was often of a very poor standard and they lacked basic provisions such as appropriate food and warm clothing.

*“I’m asking for help because I don’t know what’s going on… they kept me inside the airport…and then…they took me to the detention centre… I can’t speak English so…they always*,* always*,* always force me to speak French. I said… ‘I don’t speak French*,*’ I could speak some words but it’s not like I can say whatever I want…The Home Office… they still not believe me…. If you are telling people [and] they don’t believe you. It’s harmful…All these things I was telling them*,* but they don’t believe you …They kept me there over four months… inside that detention centre.” (Male*,* 007)*.

### The impact of PMIE exposure on wellbeing

#### The psychological impact of exposure

Participants described that their experiences of PMIEs both during, and after, human trafficking often stayed with them for several years after the event(s). The experiences reportedly often caused intense feelings particularly of shame and anger. Their difficulties with feelings of shame and low mood reportedly contributed towards difficulties with self-care following the PMIE. Several participants described finding it very difficult to move on from their experience, often struggling with intrusive thoughts and rumination. A number of participants reported difficulties with low mood or mood swings and experiences of suicidal ideation or suicide attempts.

*“Yes*,* it has an effect on my mental health because I was really*,* really down and I cannot sleep*,* I won’t eat so I was really*,* even ‘til now I’m still I’m on medication*,* I cannot sleep without medication because as soon as I get in bed my mind will just reflect to this my problem. My mind will just be how long am I going to continue like this?… How long [until I will] be free? How long [until I will] live like a normal woman?” (Female*,* 002)*.

These cognitive and emotional changes were thought to be exacerbated by feelings of loneliness and social isolation. This sense of isolation was felt to be caused by participants difficulties in feeling close to others post-PMIE due to feelings of shame and self-stigma, concerns that they could be the subject of gossip, as well as very low trust in other people following their experiences.

*“I don’t have too much friends because it’s very difficult and when I go with my daughter now to playground or playgroup it’s very hard to talk with other friends or to keep too much friends. I have three or four friends here…It’s difficult because what happened with me in the past it’s very difficult to trust now….in your mind or your heart*,* is so painful. It’s so painful to trust or to believe [someone]… it’s very*,* very hard for me.” (Female*,* 004)*.

*“I [was] always alone thinking*,* thinking*,* thinking*,* thinking*,* thinking*,* sometimes I cry*,* I cannot even eat…. because as long as I stay alone all the time [I] start thinking.” (Male*,* 007)*.

#### Alterations to beliefs about the self, world and future

Several survivors described the negative impact that their experiences of PMIEs had taken on their view of themselves, their future and the world around them. Participants often held global perceptions of the world as a dangerous place and, as they had put their trust in the wrong people in the past (e.g. traffickers), they now felt that other people cannot be trusted and it is only a matter of time before they would be let down again. Following their experience, participants described feeling permanently negatively changed, with some labelling themselves as a bad or damaged person. Participants also considered that the dreams that they had hoped for in terms of their future education or family life were now shattered and unachievable. Several participants explained that their physical health was also negatively impacted, and they now experienced ongoing physical health challenges, such as arthritis, chronic headaches, high blood pressure, which impacted their day-to-day functioning and ability to pursue goals.

*“It’s like my soul is damaged…I’m not a nice person… I just don’t want to see anybody; I just don’t want to see any man around me. I don’t want them to touch me. If a friend touched me*,* I would snap… because of my experience it actually changed who I am…In [my home country] I was a girl that’s growing up who wants to go to school. I like studying…you know*,* those are my dreams. But unfortunately*,* dreams are shattered because I follow big dream.” (Female*,* 001)*.

*“For me it’s been very bad because I was in the [hotel] and I was sick and… I have very bad with mental health and with my health because I have headache and I suffer after with…blood pressure and it’s been very*,* very bad. Sometimes I think to kill myself in those moments because it’s very difficult for me.” (Female*,* 004)*.

#### The impact of PMIE exposure on parenting responses

As a number of participants were parents, they also described how their PMIE experiences and subsequent psychological difficulties impacted their parenting responses. Parents reported considerable concerns for their children’s safety and fears that their child could experience similar abuse or victimisation. Parents encouraged their children to view the community as unsafe and other people as untrustworthy. Parents described that they had not told their children about their experiences of human trafficking or of the PMIE exposure, as their experience was felt to be too shameful to share or they were concerned it could be too distressing for their child.

*“I never tell [my son] really what happened because in my country it’s shame*,* they don’t see you as a victim*,* but they see you it’s like a mockery*,* they can mock you*,* they can see you like it’s your fault.” (Female*,* 005)*.

Most participants were single parents and described how caring for their children could be relentless and overwhelming, yet, at the same time they preferred to provide all childcare themselves rather than risk their child coming to harm at the hands of others. They accompanied their children as much as possible. Several parents reported that caring for their child helped them to focus more on the present and being a parent was felt to give their life new meaning.

*“I owe it as a duty to my kids…I owe it as a duty of care to make sure in my own best way that I can. I’ve never left my kids with anybody. Nobody…. Even my neighbour upstairs [has offered] many times*,* she’s like ‘you are stressed’…I am stressed…but I would never fall in my duty to say I want to leave them. In my head I’m like what if something goes wrong? What if? I would never*,* ever forgive myself. I don’t want anyone to molest my children. I have girls now. I don’t want anyone to take advantage of them at a young age as well.”(Female*,* 006)*.

*“I’m over protective of my daughter. I’m scared of everything. Scared of the dark. She’s in secondary school and I drop her off at the bus stop to make sure she goes on the bus. Then I wait for her at the bus stop to bring her home. So I’m just always scared that men they can just come and hurt women at any time. I know what it is*,* I’ve been there. I don’t want it to happen to anybody I know especially my daughter….I’m scared someone can just take you and put me in the van and drove it off…. She always wonder ‘why is my mum always like this?’ She doesn’t know.” (Female*,* 001)*.

#### Effect on post-traumatic growth

Perceived experiences of post-traumatic growth included the above-mentioned appreciation for their children and the meaning derived from their role as parents. Others described that their experiences of PMIEs had led to a growth or strengthening of their spirituality or religious beliefs which was a great source of comfort to them. Although their challenging experiences had caused them to question why God had allowed this adversity to occur, no participants reported a loss or diminishing of their spiritual beliefs due to their PMIE experiences and some experienced considerable social support from other members of their faith group. Many participants voiced a desire to create a better future for themselves and described experiencing a new drive to rebuild their lives, including pursuing educational opportunities and careers – such as pastoral roles or social work – in order to help others.

*“I’m trying to keep on because of my daughter… probably if I don’t have a child*,* probably if I don’t have a daughter*,* I might be thinking that it isn’t worth living. [I] live for my daughter and protect her with everything I have… I want to be somebody for her*,* I want to be a mother.” (Female*,* 001)*.

*“Sometimes my faith shakes to be honest. When I’m like ‘OK God loves me*,* why is this happening in life?’ But then again*,* I pick myself up and I remember that at the end of the day God was still there. He sees it all…At the end of the day…when you are not in control of a situation*,* you just let go and let God take over. So that’s how I’ve learnt to deal with things differently…[at] Sunday service there was a man of God…preaching…that said something that really stayed with me. He said ‘as a Christian*,* it does not mean that your life will be perfect. It does not mean that nothing bad will happen to you… God will never give you something that you cannot handle…Many a times things happen so that we’ll have a story to tell.’ There’s no human being that does not have a story to tell.” (Female*,* 006)*.

### Support for moral injury-related distress

Many participants who had experienced challenging encounters with the Home Office recounted how they had received considerable support from their solicitors. Solicitors were described as not only offering legal support and guidance (including access appropriate interpreters) but also practical support such as access to winter clothing, emergency transportation and accommodation. Solicitors also provided support in cases of mental ill health, arranging hospital access following suicide attempts as well as referrals to mental health services including the HBF.

“*[My solicitors] fight for the immigration… They did the job. One day I was try even to kill myself and then they took me to the hospital… I tried to drink bleach…So they took me to hospital…I have no family here. Always my help is from…my solicitors” (Male*,* 007)*.

Participants who did not access mental health treatment via their solicitor reported being referred by other NGOs or their GP, however psychological support for those who had been trafficked was generally considered by participants as difficult to access. The majority of participants who reported receiving talking therapy experienced it as helpful and beneficial to their psychological recovery. As many participants had not disclosed their traumatic experiences to others, they described the importance of being able to trust their therapist who was often one of the few people they had spoken about the event(s) to. This trust was built over time by creating a shared understanding that therapy was confidential and non-judgemental.

*“Yes for me it’s like shame*,* I can’t say anything about that…I don’t tell people what’s really*,* really*,* really happened to me…During the therapy…I’ve told them everything because I feel they are there for me*,* they are there for people like me and they have a confidential*,* this is their job so I need to be open to them…It’s not easy but I need to tell them. If I want a solution*,* I need to tell them the truth because they are there for people like me… they are there for that*,* they can help me… I’m not the only one*,* this organisation has so many people*,* so I need to do it.” (Female*,* 005)*.

Some participants described how on starting therapy their psychological symptoms initially worsened; however, symptoms did improve in time and participants also detailed how therapy and their therapist gave them strategies to better manage post-trauma triggers, intrusive thoughts and nightmares. Therapy was felt to help participants to better understand their experience, with some describing how they experienced a reduction in self-blame post-treatment.

*“I always think I’m still there*,* I’m still with where this is happened to me. So [my therapist] gave me the chart to put on the wall and as soon as I’m thinking like that…I will open my eye and see the picture and I will know that I am no longer there… and I’m safe… Then she told me whenever I feel angry I should just…take a deep breath and then close my eyes for…a couple of minutes*,* I will feel a bit relieved.” (Female*,* 002)*.

Other features of the HBF treatment centre that were experienced as very beneficial were less therapy-focused and more interpersonal and practical in nature. This included access to support groups with other trafficking survivors which helped participants to feel that they were not the only ones to have experienced these types of events. As many participants were socially isolated, this group support provided through the centre was experienced as incredibly valuable and an opportunity to feel like a ‘*part of a family’* (Male, 007). Group support activities did not necessarily involve discussing the PMIE, but included sharing meals together, English lessons and local sightseeing. In addition to therapy, access to practical support through the treatment centre was also experienced as very beneficial, including access to winter clothing, furniture, bedding, baby products as well as support to access medical care (e.g. writing letters on participant’s behalf to the GP) and accommodation.

*“To be honest Helen Bamber they did a lot… they supported me well. Maybe if I needed anything whatsoever or had any issues whatsoever*,* housing*,* children not having enough clothes or there is no money… there were times that year they gave us cash…they really helped me. So*,* they made a difference in my life… So being a mum they made it easy for me.” (Female*,* 006)*.

*“Helen Bamber…were very helpful. Everyone there saved my life because… they gave me therapy*,* advice and then…they took us around [the city] so many places*,* museum*,* so they keep me busy. So that was really helpful for me… so [every] week I can go there…and look like it’s part of family…If they have events*,* they will call us and we can come and join together and eat together*,* drink together. So*,* it was part of family you know.” (Male*,* 007)*.

## Discussion

The aim of this study was to explore human trafficking survivors’ experiences of PMIEs and moral injury, the impact of moral injury on wellbeing, and the factors that may influence wellbeing outcomes following PMIEs. We identified three core themes: exposure to PMIEs both during and after experiences human trafficking; the impact of events on wellbeing and daily functioning; and support for moral injury-related distress.

We found that trafficking survivors reported PMIEs which included betrayal events – such as being lied to and tricked into exploitation by trusted loved ones - and being subjected to extreme human suffering. We also found that interactions with authorities, such as police or Home Office immigration staff, could be experienced as potentially morally injurious as survivors described not being believed when asking for help or provided with adequate care or support. This presentation of index events is broadly consistent with the wider literature on moral injury [14]; for example, studies of PMIEs in refugee populations which found PMIEs to generally include transgressive acts committed by the self and/or others [34]. However, it is notable that participants in the present study did not report transgressive acts committed by themselves. This could be attributed to ongoing fear of prosecution by authorities, and/or the time taken to develop trust for sufficient disclosure. In consultation with HBF therapists’, it was described that this type of disclosure almost never happens during initial assessments, and may occur during the therapeutic process as trust develops. At the same time, this study is novel in highlighting the betrayal component of PMIEs – committed by both those in position of authority as well as trusted friends and loved ones - that can be experienced by human trafficking survivors. As many of the existing moral injury scales have been developed and validated in occupational samples (e.g. military personnel, healthcare workers) and focus on betrayal by those in positions of authority, it is important that future studies aiming to explore moral injury in trafficked survivors consider whether these measures are sufficiently nuanced to capture PMIEs in this population. The results of this study also contribute towards the existing literature highlighting the negative impact that poor treatment by immigration staff can have on survivor’s wellbeing [23, 35] and should be considered by policy makers when developing protocols for processing trafficking survivor’s cases and visa applications.

The qualitative approached utilised in the present study allowed for a thorough exploration of survivor’s beliefs and feelings following PMIE exposure. Survivors reported considerable distress, describing intense feelings of shame, low self-worth and anger. Survivors also reported substantial difficulties forming close relationships with others, due to their experiences of betrayal by trusted others as well as their feelings of shame and stigma. These responses are consistent with research conducted with both military and non-military populations [25, 36, 37], indicating that the profile of psycho-social responses to PMIEs could be similar across contexts. As moral injury related mental health difficulties can respond poorly to standard exposure-based PTSD treatments such as Prolonged Exposure [16, 17, 19, 38], these findings may therefore have implications for researchers and clinical care teams aiming to develop tailored moral injury treatments by highlighting the range of symptoms can be experienced following PMIEs. At the same time, our results also show that despite the substantial adversities faced by survivors exposed to PMIEs, they described positive outcomes including a growth in their spirituality and deriving great meaning from their role as a parent. These experiences of posttraumatic growth [39] are also consistent with previous studies examining responses following PMIEs [14, 25, 26] and highlight the need for future studies to comprehensively examine the range of outcomes and meaning that is made after PMIE exposure.

This study also adds to the limited literature about the impact of moral injury on family functioning as well as the few studies which have explored the impact of human trafficking on parenting responses [40, 41]. Survivors who were parents and had experienced PMIEs reported considerable concerns that their children may also become victim of similar abuse and encouraged them to see the world as dangerous and others as untrustworthy, often insisting on accompanying them. These parental behaviours and cognitions are consistent with previous studies of parents and families who have experienced trauma [42–45]. Children of parents with PTSD and other serious mental disorders are significantly more likely to experience emotional and behavioural problems themselves [45]. Without intervention, these children can continue to experience mental ill health into adulthood, which can have costly and devastating consequences, including worse physical health and poor social, educational and employment outcomes [46]. No participant in the present study described accessing psychological services for their child and it was beyond the scope of this study to explore the wellbeing of survivors’ children. Nonetheless, as psychological treatments offered by services are often limited in length and scope due to a lack of resources, and primarily focus on addressing the mental health symptoms of the survivor as an individual [4], these results highlight the need for comprehensive care for trafficked survivors, as well as others who experience PMIEs, that includes a focus on adaptive parenting. The added provision of family-centred support for the children of trafficking survivors may also be beneficial.

We identified a number of sources and features of support that survivors found especially helpful following PMIEs. Solicitors as well as clinicians providing mental health treatment reportedly provided survivors with a wide range of support, including legal, emotional as well as practical assistance. Notably, survivors described that the clinic’s provision of group support, including outings and English lessons, was very beneficial. This is consistent with the broader literature that social support is associated with more positive adjustment post-trauma and highlights the potential importance of comprehensive care that includes social support interventions to mitigate against PTSD [47, 48]. Nonetheless, previous studies show that clinical care teams report considerable uncertainty about how to best support patients struggling following moral injury [16, 17], whether solicitors face similar concerns and feel able to adequately support clients with moral injury-related distress is currently unclear.

This study has several strengths and limitations. A strength of the study was the qualitative methodology which allowed for a thorough examination of survivor’s lived experiences and the small sample size permitted detailed analysis of the dataset [49]. A second strength was that participation in the study was conducted remotely (e.g. via telephone) as well as being confidential and anonymous which could have helped survivors feel able to discuss their challenging experiences. Among the limitations is the opportunity sampling strategy used and it would be beneficial for future studies to explore the experiences of survivors who accessed formal support from other UK-based organisations, such as the Red Cross, to determine the generalisability of the findings. A second limitation was the lack of member checking as it was not feasible in this study to return transcripts/themes to participants. It is possible that survivors from different cultural backgrounds may experience and respond to PMIEs differently and whether these findings are generalisable to trafficking survivors in international contexts is also unclear at this stage and more research is needed.

Despite these limitations, this study makes a number of relevant contributions to the literature. First, as the majority of moral injury research to date has focused on samples working in specific occupational roles – primarily military or healthcare workers [14, 50]- these findings expand the existing literature of moral injury highlighting that human trafficking survivors may also be vulnerable to moral injury. Second, our findings indicate that survivors may experience potentially morally injurious events both during their period of exploitation by traffickers as well as their later treatment by and interactions with statutory and Government organisations. Third, the results illustrate the impact that PMIE exposure can have on survivor wellbeing and daily functioning, including parental responses. Finally, our findings highlight the sources and types of support experienced as particularly beneficial by survivors exposed to PMIEs. This could be used by clinical care teams and organisations that support trafficking survivors to shape and inform targeted guidance and support to ensure those affected by moral injury receive high quality care.

## Data Availability

No additional data are available.
